# New Insights in Laboratory Testing for COVID-19 Patients: Looking for the Role and Predictive Value of *Human epididymis secretory protein 4* (HE4) and the Innate Immunity of the Oral Cavity and Respiratory Tract

**DOI:** 10.3390/microorganisms8111718

**Published:** 2020-11-02

**Authors:** Annalisa Schirinzi, Angela Pia Cazzolla, Roberto Lovero, Lorenzo Lo Muzio, Nunzio Francesco Testa, Domenico Ciavarella, Giuseppe Palmieri, Pietro Pozzessere, Vito Procacci, Francesca Di Serio, Luigi Santacroce

**Affiliations:** 1 AOU Policlinico Consorziale di Bari—Ospedale Giovanni XXIII, Clinical Pathology Unit, 70124 Bari, Italy; schianna@gmail.com (A.S.); robertolovero69@gmail.com (R.L.); diseriofrancesca@tiscali.it (F.D.S.); 2 Department of Clinical and Experimental Medicine, Università degli Studi di Foggia, 71122 Foggia, Italy; elicio@inwind.it (A.P.C.); lorenzo.lomuzio@unifg.it (L.L.M.); nunzio.testa@unifg.it (N.F.T.); domenico.ciavarella@unifg.it (D.C.); 3 Private Practice, 70124 Bari, Italy; joseph0794@live.it; 4 AOU Policlinico Consorziale di Bari—Ospedale Giovanni XXIII, Emergency Medicine and Surgery Unit, 70124 Bari, Italy; piero.pozzessere@libero.it (P.P.); v.procacci@virgilio.it (V.P.); 5 Ionian Department (DJSGEM), Microbiology and Virology Lab, Università Degli Studi di Bari, 70124 Bari, Italy

**Keywords:** COVID-19, SARS-CoV-2, laboratory test, Human epididymis secretory protein 4 (HE4), interleukin-6 (IL-6), presepsin (PSP), procalcitonin (PCT), innate immunity

## Abstract

COVID-19 is a viral pandemic caused by the new coronavirus SARS-CoV-2, an enveloped positive stranded RNA virus. The mechanisms of innate immunity, considered as the first line of antiviral defense, is essential towards viruses. A significant role in host defense of the lung, nasal and oral cavities is played by Human epididymis secretory protein 4 (HE4) HE4 has been demonstrated to be serum inflammatory biomarker and to show a role in natural immunity at the level of oral cavity, nasopharynx and respiratory tract with both antimicrobial/antiviral and anti-inflammatory activity. Several biomarkers like IL-6, presepsin (PSP), procalcitonin (PCT), CRP, D-Dimer have showed a good function as predictor factors for the clinical evolution of COVID-19 patients (mild, severe and critical). The aim of this study was to correlate the blood levels of CRP, IL-6, PSP, PCT, D-Dimer with He4, to identify the predictive values of these biomarkers for the evolution of the disease and to evaluate the possible role of HE4 in the defense mechanisms of innate immunity at the level of oral cavity, nasopharynx and respiratory tract. Of 134 patients admitted at COVID hospital of Policlinico—University of Bari, 86 (58 men age 67.6 ± 12.4 and 28 women age 65.7 ± 15.4) fulfilled the inclusion criteria: in particular, 80 patients (93%) showed prodromal symptoms (smell and/or taste dysfunctions) and other typical clinical manifestations and 19 died (13 men age 73.4 ± 7.7 and 6 women age 74.8 ± 6.7). 48 patients were excluded because 13 finished chemotherapy and 6 radiotherapy recently, 5 presented suspected breast carcinoma, 5 suspected lung carcinoma, 6 suspected ovarian carcinoma or ovary cyst, 1 cystic fibrosis, 3 renal fibrosis and 9 were affected by autoimmune diseases in treatment with monoclonal antibodies. The venous sample was taken for each patient on the admission and during the hospital stay. For each patient, six measurements relating to considered parameters were performed. Significant correlations between He4 and IL-6 levels (*r* = 0.797), between He4 and PSP (*r* = 0.621), between He4 and PCT (*r* = 0.447), between He4 and D-Dimer (*r* = 0.367), between He4 and RCP (*r* = 0.327) have been found. ROC curves analysis showed an excellent accuracy for He4 (AUC = 0.92) and IL-6 (AUC = 0.91), a very good accuracy for PSP (AUC = 0.81), a good accuracy for PCT (AUC = 0.701) and D-Dimer (AUC = 0.721) and sufficient accuracy for RCP (AUC = 0.616). These results demonstrated the important correlation between He4, IL6 and PSP, an excellent accuracy of He4 and IL6 and showed a probable role of He4 in the innate immunity in particularly at the level of oral cavity, nasopharynx and respiratory tract. Besides He4 together with IL6 might be involved in the onset of smell and/or taste disorders and it might be used as innovative biomarker to monitor clinical evolution of COVID-19 because He4 could indicate a multi-organ involvement.

## 1. Background

Coronavirus disease 2019 (COVID-19) is a viral pandemic emerged from East Asia and quickly spread to the rest of the world caused by the new coronavirus SARS-CoV-2, enveloped positive stranded RNA virus in the order of *Nidovirales* [1,2,3,4,5].

The average incubation period varies between 7 and 14 days and 80% of patients have mild to moderate diseases with local involvement of the upper and lower respiratory tract, 15% have a serious disease, require oxygen and 5% have critical conditions (respiratory failure, septic shock and/or dysfunction/failure multiple organs) [6,7,8,9].

COVID-19 infection is a systemic disease that recognizes the throat and nose as a gateway, in which the virus finds a welcoming environment for replication. The viral replication occurs in the upper respiratory tract and then spreads throughout the body with devastating consequences [10,11].

Other symtoms are fever, cough, shortness of breath, headache, runny nose, muscle pain, fatigue, arthralgia, sputum production, conjunctivitis, diarrhea. At the level of the lower respiratory tract, SARS-CoV-2 is responsible for interstitial pneumonia which can develop into a severe acute respiratory distress syndrome (ARDS), sepsis and septic shock, until the patient dies [12,13].

This reaction would depend on a hyper-reaction of the immune system due to the overproduction of cytokines (cytokines storm/CRS cytokine release syndrome) [14,15] responsible for vascular damage with pressure drop, clot formations (MicroCLOTS syndrome: obstructive thrombo-inflammatory syndrome of the Covid-19 microvascular pulmonary vessels) until organ collapse [16,17,18].

Inflammatory cytokines cause inflammation of the pulmonary interstitium which is invaded by inflammatory cells (leukocytes and macrophages) and exudate. The scaffold thickens, becomes voluminous and prevents the alveoli from fully expanding during inspiration with a compromised gas exchange [19].

These patients have acute respiratory distress syndrome, which requires intense mechanical ventilation therapy [20]. In the worst cases, large quantities of inflammatory cytokines come out of the lungs and through the blood they are able to reach and also hit the other organs causing the multi-organ dysfunction syndrome, which drastically reduces the probability of survival [21].

The risk factors for severe pneumonia or death are 60 years or older and the presence of medical comorbidities such as hypertension, diabetes mellitus, cardiovascular disease, chronic lung disease or cancer [22].

Inflammatory storm is characterized by the release of a wide range of cytokines [23] IL-6, IL-1B, IL-1RA, IL-7, IL-8, IL-9, IL-10, granulocyte colony stimulating factor (G-CSF), granulocyte-macrophage colony stimulating factor (GM-CSF), interferon γ (IFN-γ), interferon γ-protein inducible (IP10), inflammatory macrophage protein (MIP1), MIP1A, MIP1B, platelet derived growth factor (PDGF), tumor necrosis factor α (TNF-α) and vascular endothelial growth factor (VEGF) [24].

The production of these cytokines belongs to the mechanisms of innate immunity, considered as the first line of antiviral defense essential for immunity to viruses. To date, knowledge of the specific innate immune response to SARS-CoV-2 is extremely limited: interferons I/III are considered the most important for antiviral defense, followed by cytokines, such as TNF- α and IL-1, IL-6 and IL-18 [24].

A significant role in host defense of the lung, nasal and oral cavities is played by Human epididymis secretory protein 4 (HE4) also known as WFDC2 (WAP four-disulfide core domain protein 2). It is a N-glycosylated protein primarily identified as a protein expressed in the epididymis and concerned with maturation of sperm [25]. Subsequently it has been shown in several normal tissues, particularly in the epithelium of the oral cavity, nasopharynx, respiratory tract, in the excretory ducts of major and minor salivary glands, in pituitary, in thyroid, in pancreas, in colon, in kidney, in a wide range of malignant neoplasms (gynecological and pulmonary origin neoplasm) and in reproductive tracts of both sexes, except in the ovaries, where no expression is observed [26,27].

It might function as an antiprotease like Secretory Leukocyte Protease Inhibitor (SLPI) and elafin, the other two member of the Whey Acidic Protein (WAP) domain family of proteins by protecting against proteolytic enzymes released by inflammatory cells [28].

More recently, HE4 has been demonstrated to be serum inflammatory biomarker in cystic fibrosis and to show a role in natural immunity with both antimicrobial and anti-inflammatory activity [26].

The presence of this protein in the mucous cells of the sub-mucosal glands of the upper airways, in minor glands in the nose, sinuses, posterior tongue and tonsil and in the ducts of the major salivary glands support the hypothesis that HE4 can be considered an host defense protein [26].

Several biomarkers have been suggested to identify laboratory parameters that could allow to detect early individuals that present high risk of developing severe COVID: lymphopenia, leukopenia, prolonged prothrombin time, elevated lactate dehydrogenase (LDH), elevated transaminases (AST and ALT), increased protein C-reactive (CRP) and procalcitonin (PCT), elevated levels of IL-6, CRP, LDH and D-Dimer [29].

In the early stage, white blood cell counts are normal or tend to lymphopenia/leukopenia. Some patients presented increased CRP, erythrocyte sedimentation rate (ESR), transaminases (AST, ALT), LDH, creatine kinase (CPK), myoglobin and ferritin. Serious patients presented lymphopenia, leukopenia, increase in D-dimers and thrombocytopenia that may be indicative of coagulopathies [30,31].

Costela-Ruiz et al. showed that IL-2, IL-6, IL-7, IL-10, G-CSF, IP10, MCP1, MIP1A and TNF-α concentrations are higher in ICU patients than in non-ICU patients and that IL-6, IL-10, IL-2 levels and IFN-γ gradually recover in patients who survive the disease [18].

Elevated levels of IL-6 were significantly related to taste and smell disorders [11] and to severe clinical manifestations in critically ill patients, in intensive care with respiratory failure and with poor prognosis [32].

Elevations in CRP appear to be unique to COVID-19 patients when compared to other viral infections [33]. 

Increase in D-Dimer levels and abnormal D-dimer levels are associated with poor prognosis [34,35].

According to others authors, PCT and platelet count may be potential predictors of disease severity and PCT may be considered a secondary bacterial infection marker [36,37,38].

Besides, Zaninotto et al. demonstrated that presepsin (PSP) allows during the early stages to identify COVID-19 patients with more severe disease, which will be hospitalized for a more long time [39]. PSP is a small soluble peptide generated from soluble CD14 and it regulated immune responses by interacting with T and B cells.

Xiuqu W. et al. showed elevations of serum cancer biomarkers in COVID-19 patients: squamous cell carcinoma antigens (SCC) and CA19.9 only in critical cases, carcinoembryonic antigen (CEA), carbohydrate antigens (CA-125, CA-153), cytocheretin-19 fragment (CYFRA21-1), HE4 in mild, severe and critical cases and concluded that elevations of these biomarkers is correlated with the pathological progressions of COVID-19 [40].

The aim of this study was to investigate HE4 levels in SARS-Cov-2 patients and the correlation with other inflammatory biomarkers (RCP, IL-6, PSP, PCT, D-Dimer) in order (i) to define which parameters may indicate a greater risk of developing critical forms with less chance of surviving, (ii) to identify the predictive values of these biomarkers for the evolution of the disease and (iii) to evaluate the possible role of HE4 in the defense mechanisms of innate immunity at the level of oral cavity, nasopharynx and respiratory tract.

## 2. Materials and Methods

This study was conducted from 15 March to 30 May 2020 following the provisions of the Declaration of Helsinki. The study was approved by the ethics committee of Bari (Italy) (N. 6388 COVID19 DOM protocol number 0034687/12-05-2020) and written informed consent was obtained.

Of 134 patients admitted at COVID hospital of Policlinico—University of Bari, 86 (58 men age 67.6 ± 12.4 and 28 women age 65.7 ± 15.4) fulfilled the inclusion criteria: in particularly, 80 patients (93%) showed prodromic symptoms (smell and/or taste dysfunctions) and other typical clinical manifestations and 19 died (13 men age 73.4 ± 7.7 and 6 women age 74.8 ± 6.7). 48 patients were excluded because 13 finished chemotherapy and 6 radiotherapy recently, 5 presented suspected breast carcinoma, 5 suspected lung carcinoma, 6 suspected ovarian carcinoma or ovary cyst, 1 cystic fibrosis, 3 renal fibrosis and 9 were affected by autoimmune diseases in treatment with monoclonal antibodies.

Inclusion and exclusion criteria are indicated in (Table 1).

Furthermore, the patients were divided into mild, severe and critical based on clinical manifestations (Table 1).

The venous sample, for the laboratory tests, was taken for each patient on the admission and during the hospital stay. For each patient, six measurements relating to considered parameters were performed with an interval of not less than 24 h. The sample was collected in 5 mL Vacutainer tubes without anticoagulants. Blood samples were centrifuged (1000× *g*, 15 min, 4 °C), the serum was removed and immediately stored at −80 °C until analysis. The IL-6 (v.n. 0–7 pg/mL) assay (kit Elecsys IL-6, Roche, Mannheim, Germany) and PCT assay (v.n. 0.0–0.05 ng/mL) (kit Elecsys BRAHMS PCT, Roche, Mannheim, Germany) was performed with the chemiluminescence assay using (Cobas e801, Roche, Mannheim, Germany), He4 (v.n. 0–70 pmol/L) assay (ARCHITECT HE4 Reagent, Abbott Laboratories. Abbott Park, IL, USA) with chemiluminescence assay using Architect (Abbott Laboratories. Abbott Park, IL, USA), Presepsin (v.n. 20–200 pg/mL) assay (kit PATHFAST Presepsin CARTRIDGE, Mitsubishi Chemical Europe GmbH, Düsseldorf, Germany) with chemiluminescence assay using PATHFAST^®^ Presepsin, CRP (v.n. 0–3 mg/L) nephelometric assay (kit CRP Flex reagent cartridge SIEMENS, Munich, Germany) using Siemens Dimension Vista 1500 (SIEMENS, Munich, Germany), D-dimer (v.n. < 500 µg/L FEU) assay (kit INNOVANCE^®^ D-Dimer SIEMENS, Munich, Germany) with turbidimetric assay using Siemens CS 5100 (SIEMENS, Munich, Germany).

## 3. Statistical Analysis

All analyses were performed using MedCalc program. Age, sex, symptom associated, olfactory and taste disorders are reported in numerals and percentages of the total. the mean ± SD are given using descriptive statistics for quantitative variables.

In order to evaluate which parameters may indicate a risk greater to develop severe forms and to identify predictive values of biomarkers (IL-6, CRP, HE4, PSP, PCT, D-Dimers) for the evolution of the disease, two group of patients, recovered COVID-19 (CND, COVID-19 Not-Dead) patients and not recovered COVID-19 (CD, COVID-19 Dead) patients have been identified. Besides, CND patients were divided into mild and severe and CD patients were considered critical patients. Mann-Whitney U test has been used to evaluate the differences of biomarkers levels between independent groups CND patients (mild and severe) and CD patients (critical patients). To evaluate the correlation between biomarkers in selected patients, Pearson’s linear correlation coefficient has been applied. Furthermore, Spearman’s rank coefficient correlation has been calculated. ROC curves have been used to calculate the threshold value that best discriminates between CND patients and CD patients and to derive the sensitivity and specificity of the test. It has been established AUC 0.9 to 1 as excellent accuracy, 0.8 to 0.9 as very good, 0.7 to 0.8 as good, 0.6 to 0.7 as sufficient, 0.5 to 0.6 as bad, and <0.5 as poor (useless test). *p*-value threshold of 5% was adopted for all the tests used.

## 4. Results and Discussion

Total 86 COVID-19 patients, 58 male (67.4%) (age 65 ± 13.1) and 28 females (32.8%) (age 64 ± 15.8), were admitted at COVID hospital of Policlinico, University of Bari. Table 1 shows the clinical characteristics of the patients and symptoms associated with taste and smell disorders. In all patients, smell or taste disorders occurred before the onset of COVID-19 symptoms (4 ± 1 days), while the duration of the disturbance was 21 ± 7 days.

**Figure 1:**
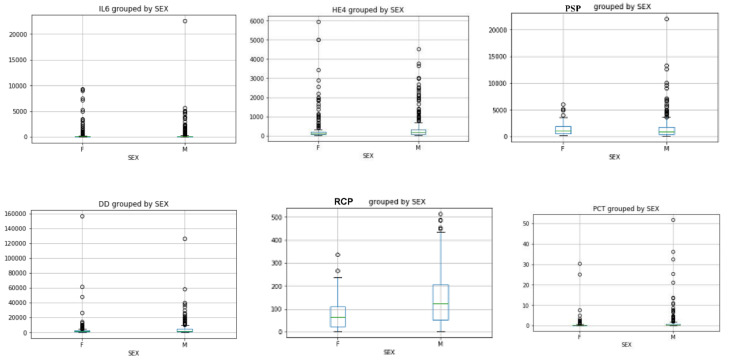
IL-6 and He4 sex distributions showed an increase in females compared to males; PSP, D-Dimer, RCP and PCT sex distributions showed an increase in males compared to females (Mann Whitney U test *p* < 0.05).

The sex distribution of IL-6 and He4 levels evaluated by Mann Whitney U test showed an increase in females compared to males (IL-6: 563 pg/mL vs. 331 pg/mL He4: 421 pmol/L vs. 382 pmol/L) (Figure 1), while the sex distribution of the values of D-Dimer (4286 µg/l FEU vs. 3856 µg/L FEU), RCP (140 mg/L vs. 76 mg/L), PCT (1.37 pg/mL vs. 0.79 pg/mL), PSP (1488 pg/mL vs. 1285 pg/mL) showed an increase in males compared to females (Figure 1).

The Mann Whitney U test, used to evaluate the values of He4, IL-6, RCP, PSP, PCT and D-Dimer provided information on differences between two independent groups (CND vs. CD). Statistically significant differences between biomarkers in CND patients versus CD patients (*p* < 0.0001) have been found (Table 2) and between mild, severe and critical patients (*p* < 0.0001) (Table 2).

Significant correlations between He4 and IL-6 (*r* = 0.797), between He4 and D-Dimer (*r* = 0.367), between He4 and RCP (*r* = 0.327), between He4 and PSP (*r* = 0.621), between He4 and PCT (*r* = 0.447) have been found in selected patients. Besides, Pearson’s linear correlation coefficient between He4 and IL-6 increased in relation to worsening of clinical conditions and it was more higher in critical patients (*r* = 0.69) while the other biomarkers did not show good correlations (He4 vs. D-Dimer *r* = 0.009, He4 vs. RCP *r* = 0.173, He4 vs. PSP *r* = 0.128, He4 vs. PCT *r* = 0.192). In severe patients He4 showed a good correlation with IL-6 (*r* = 0.48), D-Dimer (*r* = 0.346), PSP (*r* = 0.329) and no good correlations with RCP (*r* = 0.173), PCT (*r* = 0.192). In mild patients He4 showed a good correlations with IL-6 (*r* = 0.42), D-dimer (*r* = 0.20), PSP (*r* = 0.323) (Table 2).

Good correlation between He4, PSP and IL-6 led to hypothesize that these three parameters reflect the clinical course of the disease.

Spearman’s rank coefficient correlation (*r_s_*) showed an excellent correlation between He4 and IL-6 (*r_s_* = 0.70), between He4 and PSP (*r_s_* = 0.498), between He4 and PCT (*r_s_* = 0.39), between He4 and D-Dimer (*r_s_* = 0.211) between He4 and RCP (*r_s_* = 0.30) in CND group. In critical patients (CD group) He4 correlated excellently with IL-6 (*r_s_* = 0.698), with PSP (*r_s_* = 0.345), PCT (*r_s_* = 0.43), while there were no correlations with D-Dimer (*r_s_* = 0.151) and RCP (*r_s_* = −0.08) (Table 3) Significant correlations between He4 and IL-6 and between He4 and PSP in critical patients (CD group) indicated the presence of complications that induced death. In mild patients He4 showed an excellent correlation with IL-6 (r_s_ = 0.65) and a good correlation with D-Dimer (*r_s_* = 0.229), RCP (*r_s_* = 0.32), PSP (*r_s_* = 0.408), PCT (*r_s_* = 0.37). In severe patients He4 showed an excellent correlation with IL-6 (*r_s_* = 0.633), a good correlation with D-dimer (*r_s_* = 0.288), RCP (*r_s_* = 0.294), PSP (*r_s_* = 0.51) and no correlation with PCT (*r_s_* = 0.112) (Table 3).

**Figure 2:**
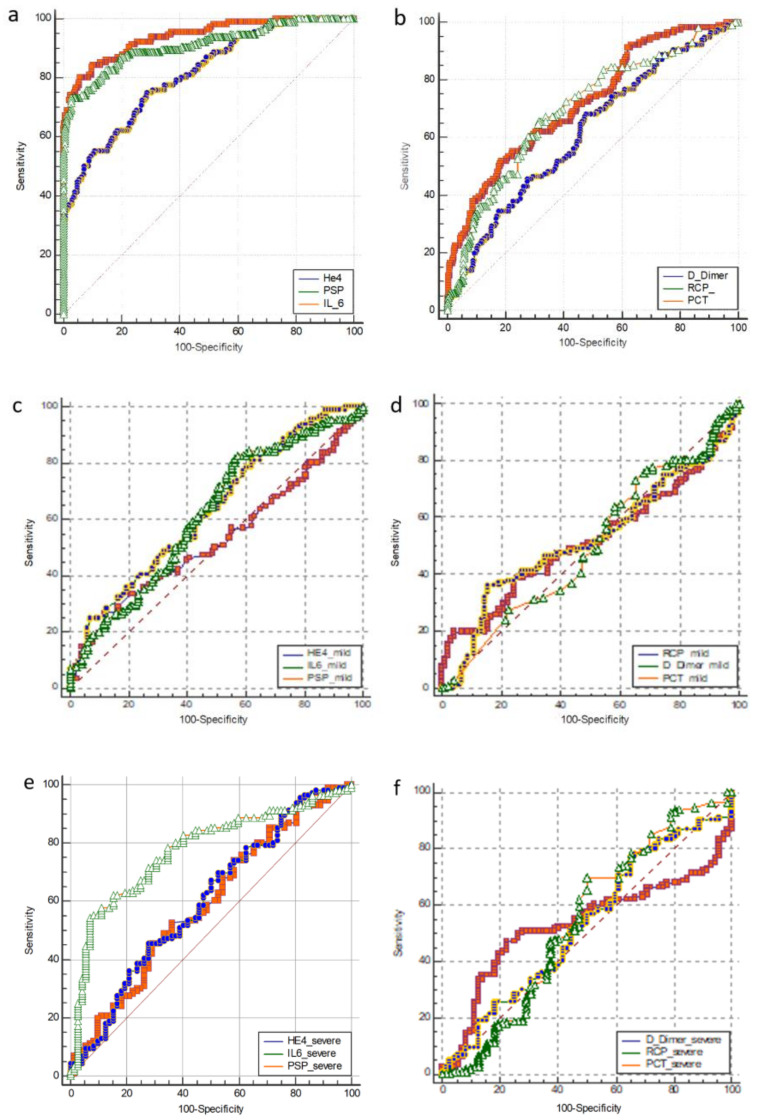
Area under curve/AUC) of considered biomarkers: in all patients (**a**) excellent accuracy for He4 (AUC = 0.92), IL-6 (AUC = 0.91), and very good accuracy for PSP (AUC = 0.81); (**b**) good accuracy for PCT (AUC = 0.701) and D-Dimer (AUC = 0.721), sufficient accuracy for RCP (AUC = 0.616); in mild patients (**c**) excellent accuracy for He4 (AUC = 0.978), IL-6 (AUC = 0.96), very good accuracy for PSP (AUC = 0.873); (**d**) good accuracy for RCP (AUC = 0.705), D-Dimer (AUC = 0.753); sufficient accuracy for PCT (AUC = 0.622); in severe patients (**e**) very good accuracy for He4 (AUC = 0.897), IL-6 (AUC = 0.851), good accuracy for PSP (AUC = 0.738); (**f**) a sufficent accuracy for PCT (AUC = 0.622) and D-Dimer (AUC = 0.6.83); a bad accuracy for RCP (AUC = 0.513).

In all patients ROC curves analysis showed an excellent accuracy for He4 (AUC = 0.92), IL-6 (AUC = 0.91) and PSP (AUC = 0.81), a very good accuracy for PCT (AUC = 0.701) and D-Dimer (AUC = 0.721) and sufficient accuracy for RCP (AUC = 0.616) (Figure 2). In mild patients. ROC curves analysis showed an excellent accuracy for He4 (AUC = 0.978), IL-6 (AUC = 0.96) and a very good accuracy for PSP (AUC = 0.873), a good accuracy for D-Dimer (AUC = 0.753) and RCP (AUC = 0.705), PCT a sufficient accuracy for (AUC = 0.622). In severe patients ROC curves analysis showed a very good accuracy for He4 (AUC = 0.897), IL-6 (AUC = 0.851), a good accuracy for PSP (AUC = 0.738), a sufficient accuracy for PCT (AUC = 0.622) and D-Dimer (AUC = 0.683), a bad accuracy for RCP (AUC = 0.513) (Figure 2).

ROC curves analysis is shown in Table 4.

Data collected showed differences between mild, severe and critical cases in terms of laboratory results: CRP, IL-6, He4, PSP, PCT, D-dimer are statistically significant elevated in critical patients (*p* < 0.0001) according to literature [29,41].

Xiang, J. et al. showed that non-survivors patients presented increase in IL-6, serum urea, creatinine, cystatin C, direct bilirubin, and cholinesterase [42].

ROC curve, used to analyze the specificity and sensitivity of different variables COVID-19 patients, indicated that IL-6, PSP, He4 reflect the clinical course of the disease and might be used to predict the evolution of COVID-19 disease.

This is the first study that correlated serum levels of He4 in COVID-19 patients with some inflammatory biomarkers (RCP, IL-6, PSP, PCT, D-Dimer). It showed a significant correlation between He4 and all the considered biomarkers in particularly between HE-4 and IL-6 (*r_s_* = 0.70) in CND patients, in mild patients (*r_s_* = 0.65), in severe patients (*r_s_* = 0.633) and in critical patients (CD) (*r_s_* = 0.698). A significant correlation was found between He4 and PSP (*r_s_* = 0.498) in CND patients, in mild patients (*r_s_* = 0.408), in severe patients (*r_s_* = 0.510) and in critical patients (CD) (*r_s_* = 0.345). These correlations led to consider that this biomarker could be used for monitoring the clinical course of COVID-19 patients together with IL-6 and PSP. Besides, it could be an indication of multi-organ involvement considering that it is produced by other organs such as pituitary, thyroid, pancreas, colon, kidney, in line with the typical evolution of the disease. In critical patients, He4 did not correlate with D-Dimer (*r_s_* = 0.151) and RCP (*r_s_* = −0.08) probably for therapeutic interferences, necessary for life support.

Therefore, these results demonstrated that He4 together with IL6 plays a predictive role in the evolution of COVID-19 disease because it showed excellent accuracy in mild patients and very good accuracy in severe patients compared to other biomarkers which showed less accuracy probably related to multi organ involvement (renal and cardiac insufficiency, thyroid and pancreatic dysfunction, etc.) and to therapies.

He4 belongs to the family of “Whey acidic protein four-disulfide core domain” (WFDC) proteins like elafin and SLPI and consists of a 42 kDa trimer, characterized by the presence of two WAP domains, respectively at the N- and C terminal ends [43].

It was hypothesized that it is involved in innate immunity at the level of human respiratory tract and oral cavity [26].

Tissue expression of the WFDC2 gene was studied in a range of healthy tissues through analysis with oligonucleotide and tissue “microarrays”. The WFDC2 gene is expressed at the level of epithelial cells of the upper airways, in the mucous cells, at the level of the ducts of submucosal glands, in minor glands of the nose, in the major salivary glands, in sublingual salivary glands and at the level of the tonsils. It was possible to demonstrate the co-expression of HE4, elafin and SLPI in the epithelium of the upper airways and of oral cavity thanks to immunohistochemical studies [26].

Physiological functions of elafin and SLPI are known and consist in the inhibition of neutrophilic elastase, serin protease and other types of proteases and killing of microbes. Furthermore, these two proteins are involved in the immune defense because they have antibacterial activity and they are able to bind the bacterial lipopolysaccharide (LPS). Besides, it was proved that SLPI is capable of blocking viral replication in vitro. On this basis it has been suggested that HE4 in physiological conditions can play an antibacterial and antiviral role in innate immune system at the level of the tract respiratory and oral cavity [26,44].

Innate immune system, which includes physical barriers such as skin and mucous membranes (oral cavity, throat and nose), various proteins and enzymes present in the tissues (defensines, lysozyme), white blood cells (macrophages, neutrophils, innate lymphoid cells, dendritic cells and Natural Killer), natural antibodies (IgM, IgG3, and IgA), MBL (mannan-binding lectin), is not specific, but it is activated very quickly within a few hours from infection and it is the first to intervene. According to some hypotheses, children would somehow be spared from SARS-CoV-2 because their innate immune response is stronger than in adults, although they still have an immature immune system [45].

Initial infection causes lyses of pneumocytes and activates multiple pathways of innate immunity through TLR, NLRP3/inflammasome activation or triggers of cytoplasmic DNA sensors such as cGAS-STING and RIG-I-MAVS. This activation drives the production of cytokines that activate antiviral gene expression programs in neighboring cells and recruit other innate and adaptive immune cells involved in antiviral immunity and tissue homeostasis [46].

Therefore, SARS-CoV-2 infection could be due to the depletion over time of the antiviral defenses linked to the innate immunity associated with a high production of inflammatory cytokines [47].

Despite this, there are insufficient data to recommend either for or against the use of any antiviral or immunomodulatory therapy in patients with COVID-19 who have mild, moderate, severe, or critical illness [48,49,50]. Researchers are carrying out incessant efforts towards understanding these topics, including on translational regenerative approaches, such as mesenchymal stem cells and probiotics [51,52,53,54,55].

Most of the typical clinical symptoms of COVID 19 patients were attributable to the presence of numerous cytokines in the bloodstream, in particular IL-6. High levels of IL-6 appeared to be involved in taste and/or smell dysfunction. The presence of a significant correlation between IL-6 and He4 led to hypothesize an important role of He4 in the pathogenesis of smell and/or taste disorders and in the mechanisms of innate immunity. To date, He4 dosage has been mainly used for the follow-up of oncological diseases (ovarian, endometrial and lung cancer), renal fibrosis and cystic fibrosis and has not been taken into consideration in the follow-up of COVID-19 disease in association with other biomarkers [56,57,58,59].

## 5. Conclusions

This is the first study based on clinical evidence and laboratory data that correlated serum levels of He4 in COVID-19 patients with some inflammatory biomarkers (RCP, IL-6, PSP, PCT, D-Dimer) showing the important role of He4 in innate immunity at the level of oral cavity, nasopharynx and respiratory tract and a possible involvement in the onset of smell and/or taste disorders in association with IL-6. High levels of He4 that significantly correlate with IL-6 and PSP have been detected in COVID-19 patients; for this reason He4 could be used as an innovative biomarker for monitoring the clinical evolution of the disease and for the pharmacological management of these patients. Further studies would be necessary to investigate the role of He4 in innate immunity in COVID-19 patients and its involvement in smell and/or taste disorders considering the limits of the samples examined (reduced number of enrolled patients, the inhomogeneity of the sample between males and females).

## Figures and Tables

**Table 1:** General characteristics, associated symptoms, associated pathologies of 67 COVID-19 patients.

Inclusion Criteria	Exclusion Criteria
- age > 18 years - laboratory-confirmed COVID-19 infection (reverse transcription-polymerase chain reaction, RT-PCR)	- patients without a laboratory-confirmed diagnosis of COVID-19 infection - patients with olfactory or gustatory dysfunctions before the epidemic (congenital anosmia, side effects of drugs, previous surgery or radiotherapy in the oral and nasal cavities, head injury, sinonasal diseases, allergic rhinitis) - patients with systemic diseases (iron deficiency, autoimmune diseases) - patients with some malignant neoplasms (ovarian cancer, pulmonary and breast adenocarcinoma, renal carcinoma, urinary tract and bladder carcinoma, oral carcinoma) - patients with cystic fibrosis - patients with neurodegenerative disorders (Parkinson’s disease, disease Alzheimer’s disease, dementia) and with major depression
**General Characteristics**	
**Gender**	**Age (years)**
Male 58 (67.4%)	68 ± 12.6
Female 28 (32.6%)	65 ± 15.1
Days from COVID-19 symptoms onset	4 ± 1
Day of duration chemosensitive disorders	21 ± 7
**Clinical classification**	
SEVERE 31 (36.1%)	65 ± 13
Male 24 (77.4%)	65 ± 13.4
Female 7 (22.6%)	64 ± 12.5
MILD 36 (41.8%)	65 ± 15.3
Male 20 (55.6%)	67 ± 12.9
Female 16 (44.46%)	67 ± 12.9
CRITICAL 19 (22.1%)	75 ± 6.9
Male 13 (68.4%)	74 ± 7.7
Female 6 (31.6%)	75 ± 6.7
**Associated Symptoms**	
Felt tired	81 (94%)
Asthenia	65 (75.5%)
Nasal Obstruction	23 (26.7%)
Small disorders	75 (87%)
Taste disorders	69 (80%)
Fever	83 (96.5%)
Cough	73 (84.8%)
Headache	45 (52.3%)
Sore throat	63 (73.2%)
Abdominal symptoms	10 (11.6%)
Muscle or joint pains	79 (91.8%)
Chest pain	70 (81.3%)
Nausea	40 (46.5%)
Vomit	39 (45.3%)
Loss of appetite	39 (45.3%)
Problems breathing	71 (82.5%)
Diarrhea	10 (11.6%)
**Associated Pathologies**	
Diabetes	31 (36.04%)
Hypertension	57 (66.2%)
Respiratory insufficiency	36 (41.8%)
Gastroesophageal reflux disease	31 (36.04%)
Thyroid diseases	21 (24.4%)

**Table 2:** Mann Whitney U test and Pearson’s correlation coefficients between considered biomarkers in mild, severe, (CND = COVID-19 Not- Dead) and critical patients (CD = COVID-19 Dead).

a	Mann Whitney U test in all patients		
Variable	*p* value		Mean value
He4 level (CND) vs. He4 (CD)	<0.0001		110 vs. 1274
IL6 level (CND) vs. IL6 (CD)	<0.0001		256 vs. 1055
RCP (CND) vs. RCP (CD)	*p* = 0.0001		119 vs. 152
PSP (CND) vs. PSP (CD)	<0.0001		727 vs. 2543
PCT (CND) vs. PCT (CD)	<0.0001		1.19 vs. 2.01
D-Dimer (CND) vs. D-Dimer (CD)	<0.0001		3117 vs. 9739
**b**	**Mann Whitney U test in mild, severe and critical (CD) patients**		
**Variable**	***p* value**		**Mean value**
He4 mild vs. He4 severe	<0.0001		91 vs. 204
He4 mild vs. He4 critical	<0.0001		91 vs. 1273
PSP mild vs. PSP severe	<0.0001		737 vs. 1234
PSP mild vs. PSP critical	<0.0001		737 vs. 3029
IL6 mild vs. IL6 severe	<0.0001		29 vs. 109
IL6 mild vs. IL6 critical	<0.0001		29 vs. 1598
RCP mild vs. RCP severe	<0.0001		81 vs. 144
RCP mild vs. RCP critical	<0.0001		81 vs. 153
PCT mild vs. PCT severe	<0.0001		0.72 vs. 0.80
PCT mild vs. PCT critical	<0.0001		0.72 vs. 2.6
D-Dimer mild vs. D-Dimer severe	<0.0001		2091 vs. 3075
D-Dimer mild vs. D-Dimer critical	<0.0001		2091 vs. 9756
**c**	**Pearson’s linear correlation coefficients in all patients**		
**Variable**	**Pearson Coefficient (r)**	**95% confidence intervals**	***p* value**
He4 vs. IL6	0.797	0.763 to 0.826	<0.05
He4 vs. PSP	0.621	0.565 to 0.671	<0.05
He4 vs. PCT	0.447	0.376 to 0.513	<0.05
He4 vs. D-Dimer	0.367	0.290 to 0.439	<0.05
He4 vs. RCP	0.327	0.249 to 0.402	<0.05
**Pearson’s linear correlation coefficients in mild patients**			
He4 vs. D-Dimer	0.2004	0.069 to 0.324	0.0029
He4 vs. IL6	0.42	0.306 to 0.542	<0.0001
He4 vs. RCP	0.144	0.01 to 0.272	=0.0323
He4 vs. PSP	0.323	0.199 to 0.437	<0.0001
He4 vs. PCT	0.043	−0.089 to 0.175	=0.523
**Pearson’s linear correlation coefficients in severe patients**			
He4 vs. D-Dimer	0.346	0.213 to 0.466	<0.0001
He4 vs. IL6	0.48	0.336 to 0.563	<0.0001
He4 vs. RCP	0.173	0.0.30 to 0.308	=0.0174
He4 vs. PSP	0.329	0.195 to 0.451	<0.0001
He4 vs. PCT	0.192	0.50 to 0.326	= 0.0082
**Pearson’s linear correlation coefficients in critical patients (CD)**			
He4 vs. D-Dimer	0.009	−0.191 to 0.173	= 0.922
He4 vs. IL6	0.69	0.581 to 0.775	<0.0001
He4 vs. RCP	0.173	0.030 to 0.308	=0.017
He4 vs. PSP	0.128	−0.054 to 0.304	=0.168
He4 vs. PCT	0.192	0.050 to 0.326	=0.008

**Table 3:** Spearman’s rank coefficient correlation (*r_s_*) between considered biomarkers in CND (mild and severe) and in critical patients (CD).

Variable	Spearman’s rank coefficient correlation (*r_s_*) in CND patients	95% confidence intervals	*p* value
He4 vs. IL6	0.70	0.65 to 0.74	<0.0001
He4 vs. PSP	0.498	0.421 to 0.562	<0.0001
He4 vs. PCT	0.39	0.30 to 0.47	<0.0001
He4 vs. D-Dimer	0.211	0.116 to 0.302	<0.0001
He4 vs. RCP	0.30	0.21 to 0.38	<0.0001
**Variable**	**Spearman’s rank coefficient correlation (*r_s_*) in mild patients**	**95% confidence intervals**	***p* value**
He4 mild vs. IL6 mild	0.65	0.520 to 0.687	<0.0001
He4 mild vs. PSP mild	0.408	0.292 to 0.513	<0.0001
He4 mild vs. PCT mild	0.37	0.249 to 0.479	<0.0001
He4 mild vs. D-Dimer mild	0.229	0.099 to 0.351	=0.0006
He4 mild vs. RCP mild	0.32	0.196 to 0.434	<0.0001
**Variable**	**Spearman’s rank coefficient correlation (*r_s_*) in severe patients**	**95% confidence intervals**	***p* value**
He4 severe vs. IL6 severe	0.633	0.352 to 0.685	<0.0001
He4 severe vs. PSP severe	0.510	0.422 to 0.589	<0.0001
He4 severe vs. PCT severe	0.112	0.0312 to 0.251	<0.0001
He4 severe vs. D-Dimer severe	0.288	0.132 to 0.401	<0.0001
He4 severe vs. RCP severe	0.294	0.0154 to 0.335	<0.0001
**Variable**	**Spearman’s rank coefficient correlation (*r_s_*) in critical patients** **(CD patients)**	**95% confidence intervals**	***p* value**
He4 critical vs. IL6 critical	0.698	0.58 to 0.77	<0.0001
He4 critical vs. PSP critical	0.345	0.174 to 0.496	=0.0001
He4 critical vs. PCTcritical	0.43	0.27 to 0.57	<0.0001
He4 cfitical vs. D-Dimer critical	0.151	−0.032 to 0.325	=0.105
He4 critical vs. RCP critical	−0.08	−0.26 to 0.102	=0.383

**Table 4:** The area under the receiver operating characteristic (ROC) curve (AUC) of the studied biomarkers and evaluation of their cut off.

All Patients						
Parameter	AUC	95% confidence intervals	Cut off	Sensitivity	Specifity	*p* value
He4	0.92	0.898 to 0.95	359	80	92	<0.0001
IL-6	0.91	0.88 to 0.934	212	73	93	<0.0001
RCP	0.616	0.573 to 0.658	88	68	52	<0.0001
PSP	0.81	0.77 to 0.84	1179	75	71	<0.0001
PCT	0.701	0.66 to 0.740	0.37	65	68	<0.0001
D-Dimer	0.721	0.680 to 0.759	3757	51	81	<0.0001
**Mild patients**						
**Parameter**	**AUC**	**95% confidence intervals**	**Cut off**	**Sensitivity**	**Specifity**	***p* value**
He4	0.978	0.955 to 0.991	198	90	94	<0.0001
IL-6	0.96	0.93 to 0.979	96	88	98	<0.0001
RCP	0.705	0.653 to 0.753	88	68	64	<0.0001
PSP	0.873	0.832 to 0.907	1179	55	94	<0.0001
PCT	0.622	0.565 to 0.677	1.07	36	86	<0.0001
D-Dimer	0.753	0.703 to 0.798	3757	51	89	<0.0001
**Severe patients**						
**Parameter**	**AUC**	**95% confidence intervals**	**Cut off**	**Sensitivity**	**Specifity**	***p* value**
He4	0.897	0.857 to 0.929	425	74.14	95.21	<0.0001
IL-6	0.851	0.806 to 0.890	212	73	94	<0.0001
RCP	0.513	0.455 to 0.542	184	34	72	<0.0001
PSP	0.738	0.684 to 0.786	2069	50	89.89	<0.0001
PCT	0.622	0.565 to 0.677	1.07	36	86	<0.0001
D-Dimer	0.683	0.628 to 0.735	5284	38.9	87.7	<0.0001
